# Optimizing DG Handling: Designing an Immersive MRsafe Training Program

**DOI:** 10.3390/s24216972

**Published:** 2024-10-30

**Authors:** Chi Ho Li, Elle Wing Ho Chow, Manviel Tam, Pak Ho Tong

**Affiliations:** 1 Department of Construction and Quality Management, School of Science and Technology, Hong Kong Metropolitan University, Hong Kong, China; 2 Department of Supply Chain and Information Management, School of Decision Science, Hang Seng University of Hong Kong, Hong Kong, China; whchow@hsu.edu.hk (E.W.H.C.); manvieltam@hsu.edu.hk (M.T.); danieltong@hsu.edu.hk (P.H.T.)

**Keywords:** mixed reality, DG handling, training program, immersive systems

## Abstract

The rapid growth of e-commerce has significantly increased demands on logistics providers, particularly in the areas of product handling and shipment. One of the most challenging and critical aspects of this process is the handling of dangerous goods (DG). This is a complex and time-intensive operation that requires safety measures and proper packaging and labelling, because mishandling DG can lead to severe injuries, property loss, and legal liability. This puts additional pressure on logistics providers to efficiently manage DG while maintaining speed and safety in the context of e-commerce. To meet this challenge, there is an urgent need to advance training programs and enhance the operational efficiency for DG handling. The use of mixed reality (MR) technology offers a promising solution. By seamlessly integrating virtual elements with real-world environments, MR has the potential to greatly improve the effectiveness and efficiency of the training of DG handling. Earlier research has examined MR in various fields, while there is still a research gap in applying MR specifically to the training of DG handling. This paper seeks to address the current research gap by presenting a novel MR model, named “MRSafe,” for a training program on the safe handling of DG. The model offers users virtual experiences and comprehensive guidance to provide operational decision support.

## 1. Introduction

The swift advancement of information technology and the development of globalization have had a profound influence on the realm of e-commerce, revolutionizing the way in which businesses engage in online transactions and conduct digital commerce [1]. From the customer’s viewpoint, e-commerce dramatically saves time and enhances the experience of placing orders from anywhere in the world at any time [2]. The convenience of the shopping experience offered to customers has contributed to increased sales of e-commerce businesses. Based on a recent statistical analysis, the global e-commerce market is expected to exceed 6876 billion USD before 2024 and is expected to achieve 8034 billion USD in 2027 [3]. E-commerce is continuing to rise, and this has led to an increased demand for efficient order fulfillment. The speed and accuracy of order fulfillment have become even more critical in the face of growing e-commerce sales. Customers expect timely and accurate delivery of products, free return logistics, changes in order, and cancellation of orders [4]. These customers’ expectations place pressure on e-fulfillment centers to optimize their processes and minimize order processing and shipping times. E-fulfillment facilities are therefore essential to the processing of e-commerce orders.

As e-commerce sales grow, there is an increased likelihood of DG being purchased and shipped through e-commerce platforms. DG encompasses a wide range of substances, including explosives, flammable items, oxidizing substances, toxins, radioactive substances, and corrosive materials [5]. This category includes everyday items, such as lithium batteries and fireworks, as well as specialized materials like dry ice and gasoline-powered engines. Despite their hazardous nature, these goods play a crucial role in various industrial sectors. Medical facilities, oil and gas operations, and petrochemical plants are examples of sectors that use DG for their day-to-day functions and processes. Some might question the availability of DG on online platform1s, though buyers and sellers must have permits to be allowed to handle DG. For instance, there is a guide to the application of a DG license, approved by the associated fire services department [6], which states that the license does not omit the need to request permission from the relevant government. Additionally, only certified personnel are allowed to handle DG, having gone through classes, training, and assessments in order to become qualified. They are also required to renew their certification periodically to keep updated with the best practices [7]. All of this demonstrates the processes and difficulties a corporation must face in order to conduct trading on DG. According to [8], the daily number of railway vehicles with DG has exceeded 8000, resulting in a total volume of nearly 180 million tons of DG per year. However, it is projected that, by 2020, the daily number of railway vehicles with DG will increase to 35,000, leading to a total volume of nearly 600 million tons of DG per year. On the other hand, it is claimed that over 1.2 million shipments of DG are transported by air annually according to the International Air Transport Association (IATA) [7]. In the upcoming half a decade, DG shipments are anticipated to increase by a substantial amount, considering that the air freight growth rate is expected to be 4.9% [9]. With the potential of leading to disastrous events, and of damaging citizens, property, and the environment [10], the handling of DG has captured the concern of the public. As the demand for DG shipments grows, so do the safety requirements. The challenges encountered by e-fulfillment centers in complying with regulations and handling DG safely are presented in this paper.

In Hong Kong, the transportation of DG must be conducted in a manner that complies with both local and global standards, regardless of the modes of transportation. Due to the special nature of DG handling, people who handle the DG should be well-trained professionals in order to prevent accidents from happening [11,12]. In Hong Kong, individuals who handle DG must undergo specialized training courses and obtain the necessary certificates before they can begin working in this field. According to a DG ordinance (Cap 295) published by the government of Hong Kong (HKSAR), DG is classified into 10 categories, and all DG are controlled and managed with strict guidelines [13]. The regulation requires professionals to adhere to guidelines for labeling, packaging, and implementing safety measures during the production, storage, and transportation of DG. Ever since the onset of the COVID-19 outbreak, online shopping has become the main channel for consumption, driving a huge inflow and outflow of DG shipments locally and internationally [14,15]. The growing volume of DG handling has significantly increased the time demands and pressures associated with DG management processes. This escalation has created a pressing need for advanced training programs and improved operational efficiency in the handling of hazardous materials. These issues have inspired this study to investigate the potential of mixed reality (MR) technology as an innovative solution for enhancing DG handling training. The primary objectives are to increase practitioners’ handling efficiency and prevent serious accidents through the implementation of MR-based training programs.

MR has garnered significant interest as a means by which to enhance operational performance. As technology progresses at a breakneck pace, MR applications are finding ever more real-world applications across various industries, including engineering, business, healthcare, education, occupational safety, etc. [16,17,18,19,20]. MR is a technology that includes various components to create an immersive experience, through the creation of an environment that combines reality and the digital world [21]. With technological innovation in recent years, the interaction between users and objects can be improved due to better networks and equipment [22]. For instance, an application is developed for utilizing MR technology to enable workers to manage bridge inspection and maintenance without visiting the site in person [23]. It also enhances the efficiency of the process by excluding the handling of physical documents as the necessary documents are instead uploaded to the cloud. Another example would be supporting laboratory lectures through an MR application that omits limiting factors such as the deficiency of staff and safety concerns [24]. 

This study takes advantage of this trend by implementing MR technology in DG handling training. Thus, “MRSafe,” a model that encompasses MR technology to provide decision support concerning the handling of DG, is proposed. Users initiate the checking process by scanning the DG label with the MR device. Appropriate safety measures regarding the shipment are displayed as a checklist on the display as a reminder. After confirming the checklist, the device guides the user to proceed with the check process by going through the standard procedures. Then, packaging requirements are presented on the screen to assist users in preparing the shipment for the outbound process. Should any accident happen, like leakage and spillage, users can report the incident and call for support via the “emergency button” that always stays on the display regardless of the progress. 

The organization of this paper is presented as follows. Firstly, Section 2 outlines the methodology details to create the MRSafe model, which aims to improve DG handling performance. Experimental results and the findings of MRSafe are presented in Section 3, while Section 4 covers the discussion. The last section concludes this study. 

## 2. Methodology

A description of the MR device used is provided in this section, along with the specifics of the developed application. To illustrate the process of the proposed model, Class 4 DG is chosen as an example, given its intricate handling process involving two subclasses [13]. The workflow is presented here to illustrate the software architecture as is a table of configurations for hardware design. 

### 2.1. Software Development for MRSafe

In this study, Unity is employed as a tool to aid in the development of MRSafe. Unity’s versatility extends beyond game development and can also be utilized for producing movies and animation cinematics, as well as designing virtual environments for architecture and engineering purposes [25].

The Mixed Reality Toolkit (MRTK) created by Unity Technology, an American company focused on video game software development and headquartered in San Francisco, is a versatile programming toolkit designed specifically for Unity. It was instrumental in the development of MRSafe [26]. 

#### 2.1.1. Vuforia Engine for Image Scanning Function in Unity

**Figure 1:**
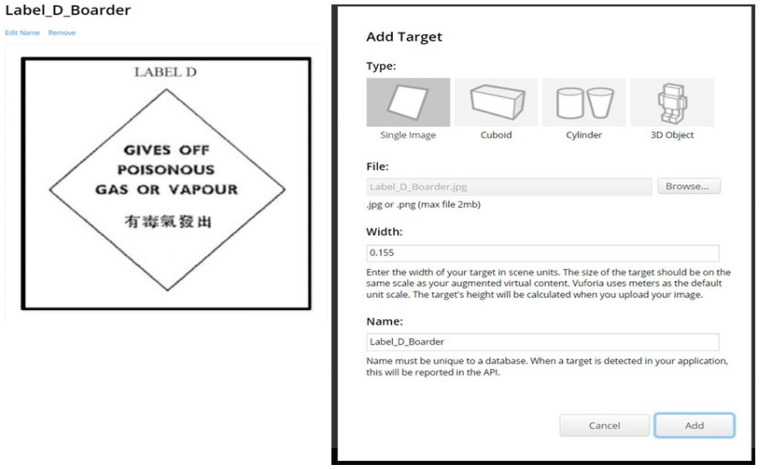
Setting up the Vuforia image database with Label D.

To incorporate the scanning function in Unity, Vuforia is utilized as a support tool. Vuforia serves as a versatile platform for creating AR and MR applications. It offers features such as application testing, allowing for the evaluation and validation of AR and MR systems in real-world environments [27]. Vuforia Engine has the ability to detect and track image targets, which are pictures that serve as reference points [28]. Image targets can be created using the Vuforia Target Manager, which supports grayscale image files like TIFF, JPG, and PNG [29]. For this case, the chosen image target is the Label D image for Class 4 DG in Hong Kong. This image is uploaded to the Vuforia database, as depicted in Figure 1. Once the photo is successfully uploaded, the logo scanning function can be activated by downloading the generated Vuforia database and integrating it into Unity. By following these steps, Unity can be equipped with scanning functionality, enabling the recognition, and tracking of specific images or logos within the Unity environment [30].

#### 2.1.2. User Interface Design for MRSafe via Unity

By utilizing the scanning feature offered by Vuforia Engine, users have the ability to scan the Label D photo in real-world settings, which activates the proposed user interface within the MRSafe program. 

**Figure 2:**
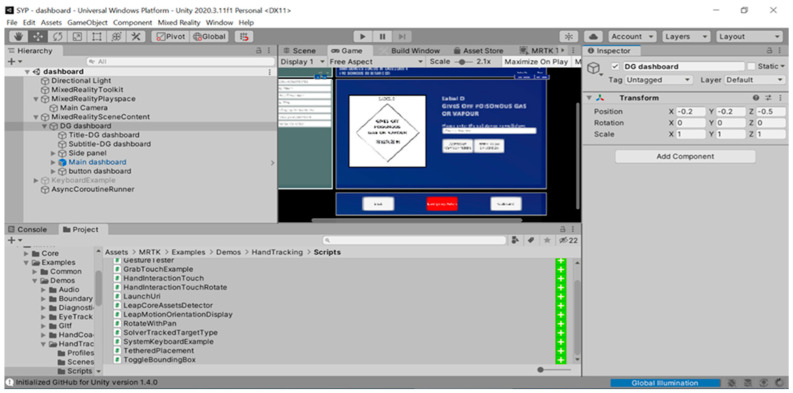
Interface of Unity when designing the proposed MRSafe program.

The user interface of the proposed MRSafe program is designed using Unity, as shown in Figure 2. The interface features a “hierarchy” panel on the left-hand side, which organizes objects in a nested manner. The “DG dashboard” object encompasses all of the significant content of the training program and serves as the parent object for the “Main dashboard.” The “Inspector” panel on the right, allows for the application of various features to the objects. One such feature is “Transform,” which allows users to assign position, rotation, and scale values to the object by configuring the position, rotation, and scale of the DG dashboard objects on the right-hand side. This includes parameters such as positions x, y, z, width, and height.

**Figure 3:**
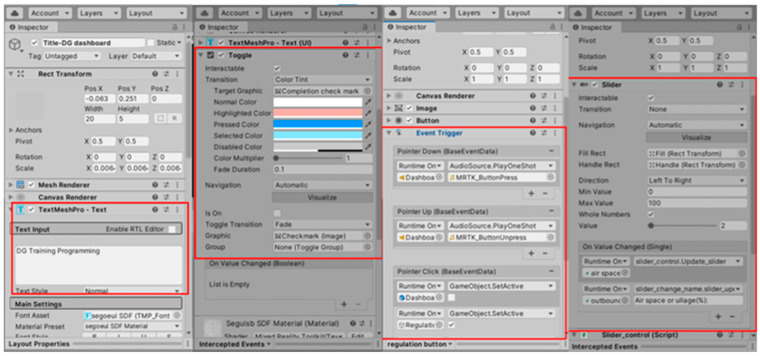
Diverse functions for the MRSafe program UI using Unity.

Additionally, several functions of Unity are incorporated in order to enhance the user experience of MRSafe, such as TextMeshPro, toggle, button, and slider, as shown in Figure 3. The TextMeshPro feature in MRSafe allows the message “DG Training Programme” to be shown on the program interface. The toggle feature creates a button that, when clicked, displays a tick mark, serving as a checklist function. The button feature enables user interaction with objects. By setting an event trigger, actions can be triggered when the button is clicked. The slider feature allows users to input and adjust values in a flexible manner. In this case, it is linked to the text referring to the airspace of the substance’s ullage. Additionally, a functionality can be implemented to evaluate the input value. For example, if 2% of airspace does not comply with regulations but 5% does, a cross mark will appear on the side panel. This value will also be stored and displayed on the “Outbound” screen for final checks and records.

### 2.2. Software Architecture of MRSafe 

**Figure 4:**
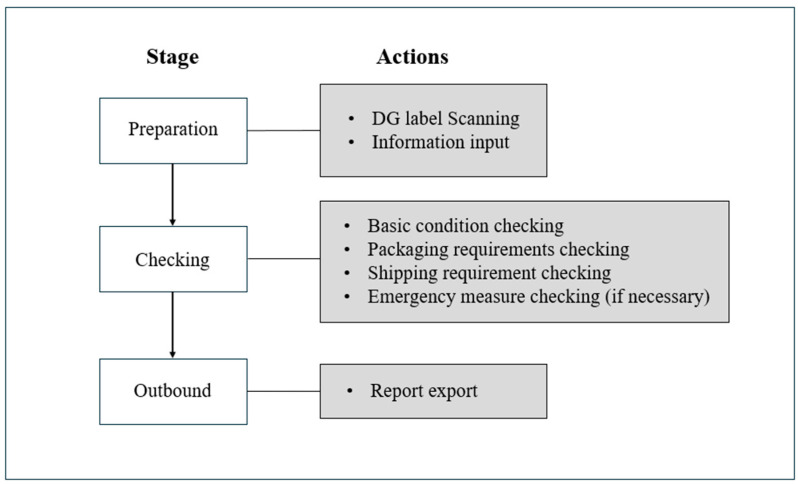
Software architecture of MRSafe.

The software structure of the proposed model can be referred to as the designed workflow for the MRSafe, and is depicted in Figure 4. There are three stages, namely preparation, checking, and outbound, with each responsible for different actions that guide users in completing the DG handling training. These three stages act as the model components, interacting with one another throughout the process. The details of each individual action are explained in the following as we elaborate the MRSafe workflow.

With reference to the general DG handling workflow, we apply MR technology in the proposed model to create an immersive environment for users to undergo training. The following illustrates a scenario of a freight forwarder that handles DG shipments and how MRSafe is designed to provide effective training.

**Figure 5:**
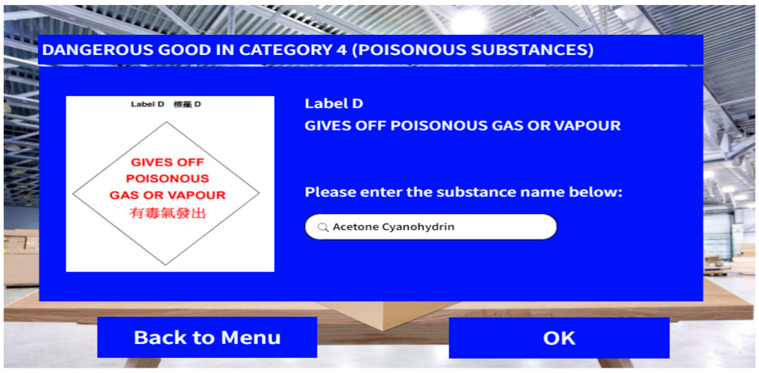
UI design––preparation.

Users will be guided to the preparation stage at the beginning. The preparatory procedure begins with the scanning of the DG labels on the shipments, followed by manual input of the substance name stored in the shipment. Specific safety precautions will be shown on the MR display in applicable cases to instruct users in handling the DG shipment. After checking each designated requirement, users are required to put a tick in the checkbox which triggers the system checking components. Should the requirements on the checklist be satisfied, the user will be navigated to the checking stage. Figure 5 illustrates the UI design of the preparation interface of MRSafe. 

**Figure 6:**
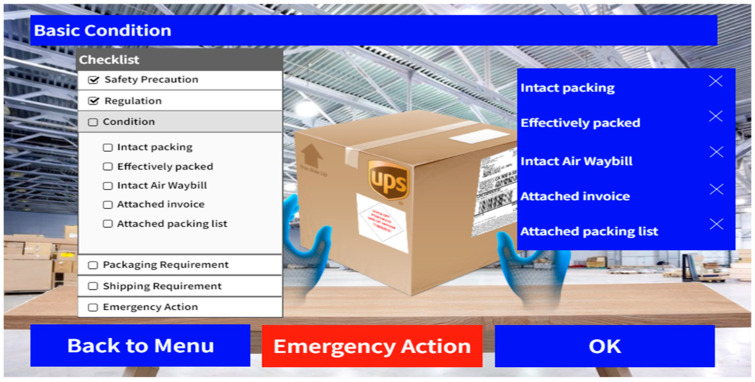
UI design––basic condition checking.

The checking stage consists of four parts, which include basic condition checking, emergency measures checking, packaging requirements checking, and shipping requirements checking. The first part refers to examining the outer condition of the shipment and the shipping documents. The ideal scenario is observing intact packaging and integrity in the documents attached to the shipment. A checklist of requirements should be completed by the users by selecting the checkbox on the list. If a condition is not fulfilled, users can put a cross in the checkbox for recording, and then a pop-up message of “Condition not fulfilled yet” will be displayed. Compared with traditional DG training, where users need to constantly refer to the materials for guidance, the checklist provides a more effective instruction method by reminding them whether a requirement is satisfied or not. Figure 6 displays the UI design of basic condition checking with a checklist on the left-hand side.

**Figure 7:**
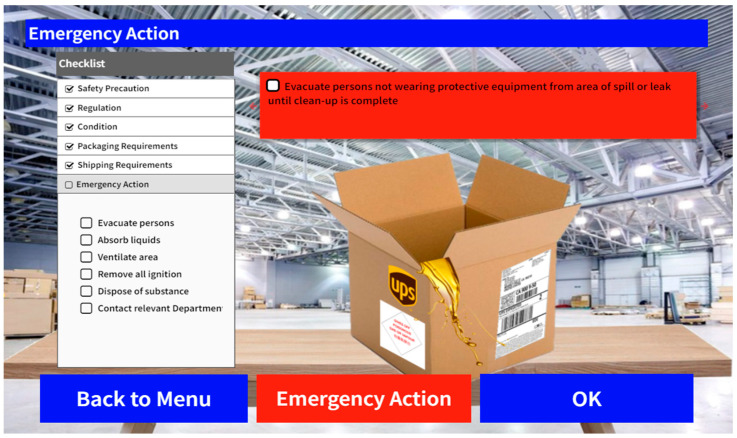
UI design––emergency measure checking.

The next component concerns itself with accidents. Whenever the situation calls for emergency measures, such as spillage or leakage of chemicals, users can press the “Emergency Action” button and switch to emergency mode. A list of emergency actions is recommended to guide users in handling the accident. Traditionally, without the list of recommended actions, users must go through the materials to find the best actions for the situation. However, with the aid of the recommendations list, a quick reference guide is provided, ensuring prompt and proper response to the problem. Figure 7 demonstrates the case of emergency; the recommended actions are listed both in the red box and in the checklist on the left. 

**Figure 8:**
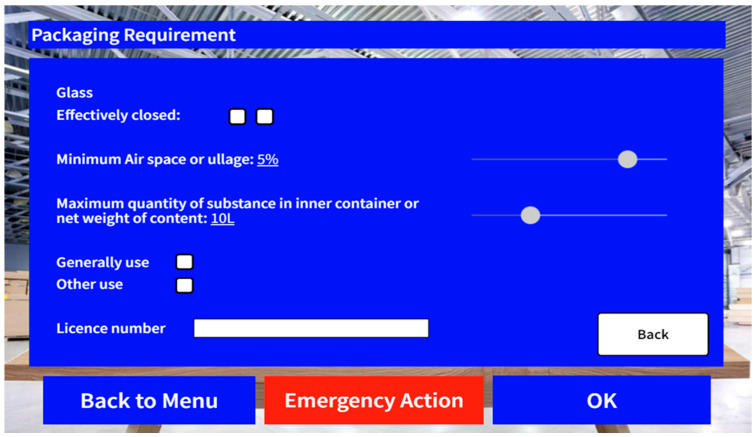
Screen capture of the slider function.

The third part focuses on checking the inner package of the shipment. For shipments that require repackaging and random checking on the inner package, users are required to select the types of containers that will be used for outbound shipments, as different containers demand varying packaging requirements in terms of, for instance, the minimum ullage, the maximum quantity, or weight of the shipment. In this case, a function called “slider” in MR devices is adopted to assist users in data input, which helps in avoiding data that are out of feasible range. Figure 8 showcases the interface that involves the slider function for better illustration. Again, similar to the previous stages, when all of the packaging requirements are satisfied, the user will be directed to the last component of this stage.

The final part of the checking stage involves the shipping requirements, which serve as the last inspection before outbound shipment. This component emphasizes the outer packaging of the shipment, which requires users to put a tick in the checkbox after satisfying the corresponding requirement. If the shipment is found to be seriously damaged in any part of the checking stage, a problem report will be generated by the system and sent to the origin for notification. It will also be stored automatically for future reference, and the concerned shipment will be repackaged and put aside until further updates. Shipments that fulfill all requirements will proceed to the outbound stage.

**Figure 9:**
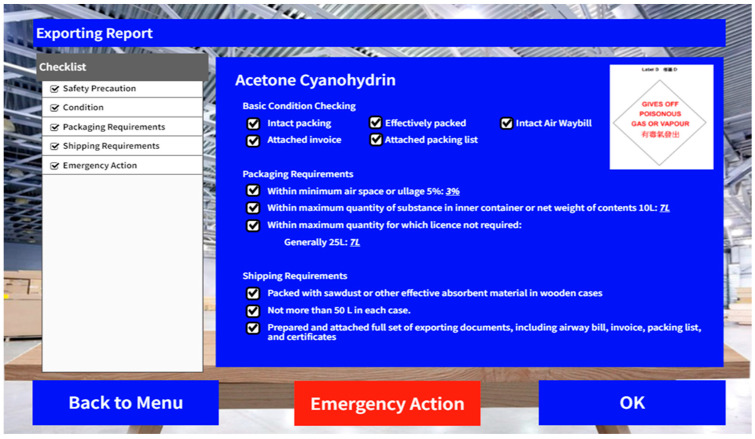
UI design––export report.

Upon reviewing and checking all of the requirements in the previous stages, the shipments are prepared for export. Based on the recorded information, export reports will be generated and stored in the system for reference. Figure 9 presents the report of a shipment that is ready for export.

### 2.3. Hardware Design of MRSafe 

The hardware configuration of the MRSafe is provided in Table 1, which lists the specification of the setup used in the experiment. Specifically, we have aimed for a 90 Hz framerate to provide an immersive and comfortable experience for users without causing dizziness or motion sickness.

In this study, the MR device was chosen as the selected MR-HMDs. This device incorporates an advanced holographic processing unit (HPU 2.0), which allows for live computer vision with low power consumption [31]. Through the use of a transparent holographic lens, virtual objects are projected to appear in a way that makes them seem like they are present in the physical world, creating a smooth integration of the virtual and tangible world. The MR device does not require any external connections as it is fully wireless and self-contained. It also includes sensors and cameras, enabling the tracking of hand-eye coordination for precise control over manual operations and delicate finger movements [32]. The training program can be effectively showcased by integrating the Unity program with the MR device. The use of MR features, such as sliders and virtual keyboards, simplifies the process of checking various packaging and shipping requirements. Moreover, the system automatically generates problem reports and export reports, ensuring that users are informed about the fulfillment of shipment requirements.

Once the MRSafe program has been developed using Unity, it can be transformed into a mixed reality (MR) program, as the goal of the study is to develop an MR training program specifically designed for handling DG. To export the DG training program from Unity into the MR device, the Mixed Reality Toolkit package (MRTK v2) is adopted. The MRTK v2 is a freely available project that offers a collection of components and functionalities to expedite the creation of applications for MR devices, supporting the MR device’s new hand tracking and eye tracking inputs [33]. 

In summary, the MRSafe model consists of the above elements, namely the hardware, software, and workflow, which create a safe and efficient approach to train users in handling DG. To illustrate the effectiveness of the MRSafe model, we have conducted an experiment to observe how it enhances DG handling training. The details are explained in the following section. 

## 3. Experiment Methods and Results

This section displays the setting and the outcomes of the experiments carried out to evaluate the proposed model. A total of 35 university students with domain knowledge in supply-chain management were initially invited to participate in the study. We consulted experts’ opinions on whether the lack of relevant professional experience would be concerning, and they concluded that the result is reasonable as the students have received lectures on basic DG handling. The invitations were conducted through face-to-face interactions to ensure clear communication and to address any immediate questions or concerns. During the experiments, students with the following conditions were excluded from the study: a history of heart illness, neuropsychiatric disorders, current use of medications, and pregnancy or planning to become pregnant. Of the 35 invited students, 30 agreed to participate in the experiment. The final sample consisted of 12 females and 18 males, with a mean (M), ± standard deviation (SD), age of 20.89 ± 1.36 years. This experiment utilized the pre-test and post-test design to evaluate the effectiveness of the MRSafe training program for handling DG. In the pre-test stage, participants were instructed to perform the handling process for Class 4 dangerous goods using traditional manual methods. In the post-test stage, participants were asked to complete the same tasks using the MRSafe on the MR device. By comparing the results from these two stages, the impacts of implementing MR technology in DG handling processes can be accessed. 

**Figure 10:**
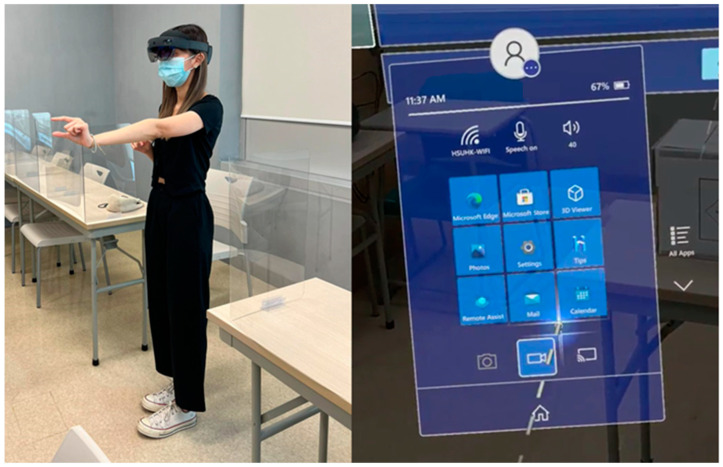
The situation of participants using MR devices for the experiments.

To initiate the training process, three basic prerequisites must be fulfilled: (1) a stable network connection, (2) being logged into the account of the MR device, and (3) a secure and open area. Furthermore, participants are requested to switch to contact lenses for the duration of the experiment if they wear glasses, in order to facilitate the use of the MR device. Following are the user settings, such as language selection and Iris set-up, which calibrates the optical parameters of the MR device according to each user. The optimal performance of the model relies on the careful execution of the setup process. Participants were tasked with performing the handling process of Class 4 DG both manually and using MR technology to evaluate the efficacy of MRSafe. Along with receiving thorough instructions, the participants were given plenty of opportunities to become acquainted with the MR setup. The participants underwent experimental procedures while standing. The individuals were asked about their thoughts and overall experiences of MRSafe after the measurements were finished. Figure 10 reveals the situation of participants using the MR device for the MRSafe experiments. 

Table 2 illustrates an overall comparison between MRsafe and the traditional manual technique. The findings show that DG training has become substantially more effective and efficient as a result of deploying MRSafe. In the case study, the introduction of MRSafe resulted in a remarkable 43.83% reduction in the duration of training sessions and a notable 7.88% decrease in the error rate of the training sessions. Further details regarding these improvements are provided in the subsequent sections. By utilizing the scanning function in MRSafe, the MR device automatically recognizes the DG label and determines the class of the DG. This eliminates the need for participants to manually classify the labels based on their own knowledge. Instead, they only need to enter the substance’s name, streamlining the process significantly. Consequently, there has been an enhancement of 4.87% in the accuracy of the DG distinguishing process. 

Moreover, there is a substantial improvement in the checking process including emergency measures checking, basic condition checking, packaging requirements checking, and shipping requirements checking. During emergency situations involving spilled or leaked DG substances, participants who are not familiar with the process in the traditional setting may need to seek assistance from staff or rely on internet resources to determine the appropriate course of action. On the other hand, MRSafe offers immediate decision support to participants during emergency situations, training users to become more efficient in finding critical information and thus making quicker and more accurate decisions. By pressing the emergency button in MRSafe, users can receive guidance such as a suggestion to evacuate individuals with appropriate protective gear from the contaminated area until the cleanup is finished. Participants can be trained to respond to emergencies more quickly with these features, resulting in a 64.91% improvement in emergency response time. For basic condition checking, participants rely on their own knowledge and experience to determine if the packing of the DG is acceptable or not in the traditional method. However, with the decision support provided, participants are guided on what to check, including intact packing, airway bill, invoice, and more. This results in a significant improvement in the efficiency of the basic condition checking process, reducing the duration by 44.56%. In the process of checking packing requirements, participants are required to determine whether the main and inner packing are made of glass, earthenware containers, or steel drums. In the traditional method, participants need to rely on their memory to recall these options. However, in the MRSafe method, participants are provided with these three options, making it easier for them to decide. The improvements in the MRSafe method are minimal compared with the traditional method with 33.54%, as it does not offer significant support to participants. In terms of checking shipping requirements, participants in the traditional method need to manually compose an exporting report, based on the judgment and information gathered from the process. On the other hand, the MRSafe method assists users by recording the information for participants and requesting their confirmation. The MRSafe approach offers a notable improvement in this process by 54.86%. Overall, the familiarity with DG handling tasks also contributed substantially to the improvement across various processes, as students recognized the similar process while using MRSafe, resulting in greater efficiency.

To provide further support for the experimental conclusions, a survey was conducted among the participants who had experienced DG handling training in both the traditional method and MRSafe. Table 3 lists the survey results, which, in accordance with the experiment result, show favorability towards MRSafe. Participants are required to answer the questions on a scale from 1 to 5, with the higher being the better and vice versa, though conversely, for user fatigue, the higher the score, the worse it is. Results show that participants are more effective and confident while using MRSafe, as it recorded 4.7 and 4.5 in respective questions while the manual method only recorded 3.1 and 3.3, respectively. They are also less tired after training, as suggested by the way in which the manual method scored 3.8 in user fatigue, while MRSafe only scored 2.5. Students also ask much fewer questions during training in MRSafe, averaging only 1 question per participant, whereas students ask 5 questions on average per person for the manual method. This is due to the lack of support in the manual method. In addition, students using the manual method experienced frustration each time they were forced to request help, which explains the far lower user satisfaction.

We have also compared our work with existing research to differentiate MRSafe from other similar models. Ref. [34] has developed a DG identification system that has been experimented with using police academy students. The system consists of three projects that educate users through virtual reality in DG observation, comparison, and case studies. Compared with our proposed model, this system features more complicated scenarios and less support to provide a more in-depth training, while MRSafe offers more assistance to users such as via the emergency mode and the automatic reporting, increasing the training efficiency. Ref. [35] aimed to create a virtual simulation of dangerous areas intended for DG in a port terminal, allowing logistic operators to undergo training. Similarly, this model serves to provide a training ground for users with minimum support, along with KPIs implemented to measure the performance of users, relying heavily on their own knowledge and skills. This might lead to less efficient training when compared with MRSafe, in which a to-do list is always on display to guide users to navigate different processes effectively.

**Figure 11:**
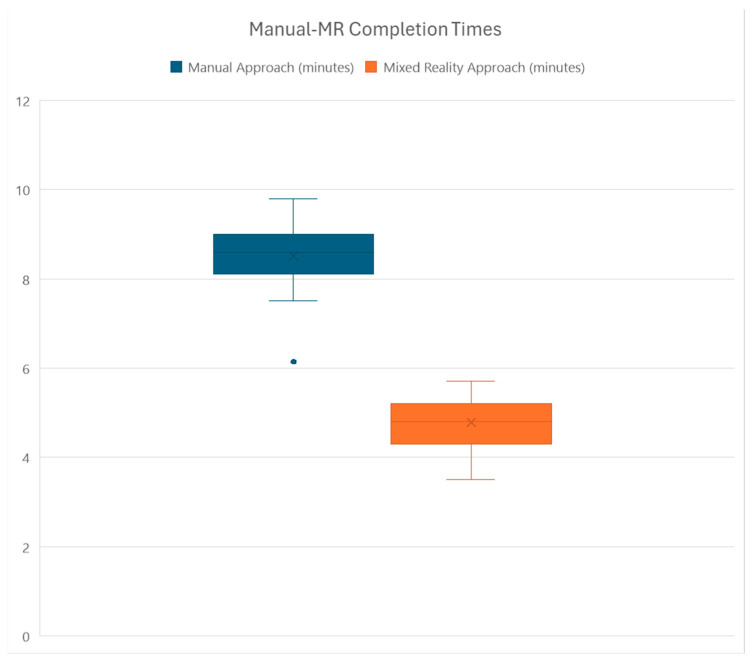
Boxplot for completion time between the manual and MRSafe approaches.

A paired *t*-test was conducted to compare the completion times of 35 students using both the manual and MRSafe approaches. The purpose of the *t*-test is to determine if there is a significant difference in the average completion times between the manual approach (mean = 8.51 min, SD = 0.69) and the MRSafe approach (mean = 4.78 min, SD = 0.62). The 95% confidence interval for the difference in means was between 3.38 and 4.08 min. Figure 11 presents a boxplot comparing the completion times of two different approaches. It shows that the completion time for the MRSafe approach is lower than that of the manual approach, indicating that MRSafe is a more efficient method. The results prove that the mean difference was statistically significant between the two approaches, as the t = 21.72 and *p* < 0.001. The standard deviation is 1.02, whereas the standard error of the mean is 0.17, suggesting a high confidence in the result. 

In addition, the effect size (*d*) for the difference in completion times between the two approaches is 3.67, with a 95% confidence interval from 2.74 to 4.60. Such a large effect size implies that the MRSafe approach is capable of substantially reducing the completion times of training.

**Figure 12:**
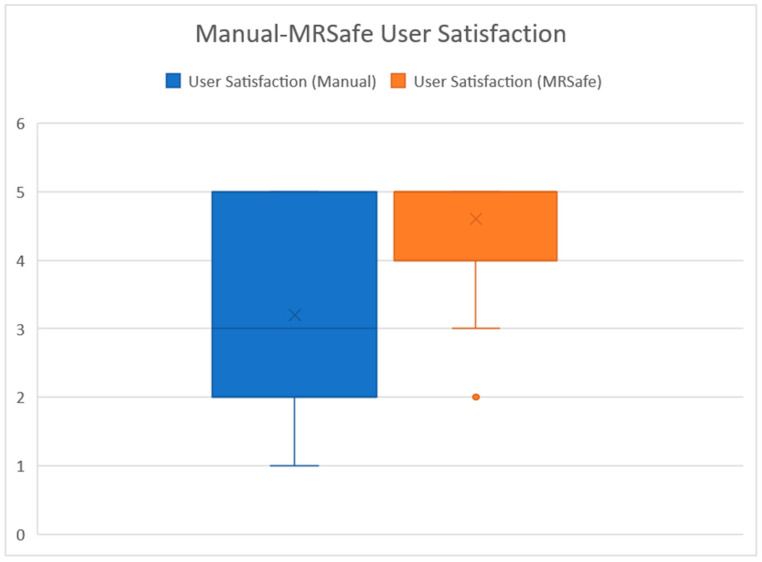
Boxplot for user satisfaction between the manual and MRSafe approaches.

For the questions related to user satisfaction regarding the MRSafe, a paired sample *t*-test was run for the survey scores of both approaches. The aim was to determine if there is a significant difference in the average user satisfaction between the manual approach (mean = 3.2, SD = 1.47) and the MRSafe approach (mean = 4.6, SD = 0.70). The 95% confidence interval for the difference in satisfaction scores is 1.77 to 1.03, supporting the observation that the manual method leads to lower satisfaction. Figure 12 presents a boxplot comparing the user satisfaction of two different approaches. The MRSafe approach shows a slightly higher satisfaction level than the manual approach, indicating a marginal improvement in user satisfaction. The MRSafe approach also exhibits less variability, suggesting more consistent user experiences. The results indicate a statistically significant difference in satisfaction levels between the two approaches with t = 7.60 and *p* < 0.001. On average, user satisfaction of the manual approach was 1.4 points lower than that of the MRSafe approach. The standard deviation is 1.09 and standard error is .18, suggesting a high confidence in the result. 

In addition, we have found the effect size (*d*) for the difference in user satisfaction between the two approaches to be 1.28, with a 95% confidence interval from 1.73 to 0.83. This suggests that the MRSafe approach has a significant positive impact on user satisfaction when compared with the manual approach. Table 4 summarizes the results of the *t*-tests.

## 4. Discussion and Implications 

### 4.1. Participants Feedback

Based on user feedback, we examine the efficacy of the application in this section. Following the experiment, a significant number of participants expressed their satisfaction and enjoyment with the application for several reasons. Participants found the image scanning functionality impressive, as it facilitated a smoother and more efficient experience. Additionally, the program’s user-friendly interface and intuitive design played an important role in participants’ satisfaction, facilitating quick navigation inside the application. Furthermore, the emergency function provided participants with quick support, instilling a sense of safety and reassurance during emergency situations. One area of criticism regarding the application was the processing power of the MR device. Users reported experiencing a slight delay and overheating issues after prolonged use. Furthermore, though the content viewed by participants wearing the MR device can be shared and viewed on computers, there is a noticeable delay between the participants’ screens and the view on the computers. As a result, this delay in sharing content between participants and computers leads to an interruption in providing instructions before experiments begin and hinders real-time collaboration to address issues as they arise. Moreover, participants recommended including photographs of the main and inner packaging in the process of checking packing requirements. This allows them to make decisions more quickly and efficiently.

### 4.2. Implications of the Proposed MRSafe Model

The proposed novel MR model, MRSafe, holds significant academic significance as it addresses a research gap in the limited number of MR applications in the field of DG handling. Valuable insights can be harnessed and added to the existing knowledge base by conducting studies to explore the application of MR technology that can be applied to improve DG handling. As such, we introduce MRSafe, a novel MR model for training programs that prioritize the safe handling of DG, offering a unique approach to improving training outcomes in this critical area. The academic importance of this research stems from the contribution to the comprehension and utilization of MR technology in the field of DG handling. This study adds to the existing literature on MR technology and also opens up opportunities for further exploration of MR technology in other domains associated with DG handling or its related disciplines.

On the other hand, several managerial implications were found in the implementation of MRSafe. Through streamlining repetitive processes and providing decision support, MRSafe optimizes training efficiency, leading to time savings and heightened productivity. Additionally, implementing MRSafe leads to significant cost savings as it minimizes the need for manual guidance during DG training. Furthermore, standardizing DG handling training ensures consistency, and reduces the likelihood of errors or confusion across departments regarding DG treatment. Moreover, the implementation of MRSafe enhances the safety culture within an organization by promoting the best practices and assures that employees receive comprehensive training in DG handling. In addition, the development stage of MRSafe offers a valuable conceptual framework for practitioners who are interested in taking the further step of integrating MR technology into practical DG handling operations. By following the roadmap presented in this study, practitioners can easily navigate the complexities of integrating MR technology into DG handling training, ensuring a smooth development process.

## 5. Conclusions

The contribution of this study is in its development of MRsafe, a model by which to improve the efficacy of DG handling by providing decision support through image scanning and information support in MR. University students with supply chain management majors were involved in the testing of MRSafe, and the effectiveness is discussed. Users scan the DG label with an MR device to initiate the checking process, which then, as a reminder, generates a checklist of recommended safety measures for the shipment, shown on the MR display. Once the checklist is verified, the device guides the user through the standard procedures for DG handling. Packaging requirements are also presented on the screen to assist users in preparing the shipment for the outbound process. Additionally, the MRSafe method keeps a record of the information entered by users and automatically generates export reports to reduce manual effort. In the event of an accident, such as chemical leakage or spillage, users can report the incident and seek support using the “emergency button” that is constantly visible on the display. The MRSafe system in this DG training application offers valuable decision support and streamlines repetitive processes for users, allowing them to handle the DG checking process efficiently and safely.

The results of the paired *t*-tests indicate that the MRSafe approach significantly out-performs the manual approach in both completion time and user satisfaction. Participants completed the training 3.73 min faster on average using MRSafe, with a very large effect size (*d* = 3.67). Furthermore, the MRSafe approach led to significantly higher user satisfaction scores, with a medium effect size (*d* = 1.28). These findings suggest that MRSafe is a more efficient and satisfying method for training compared with the traditional manual approach.

This research has several limitations, the first of which is the small sample size. The study included only 30 university students with domain knowledge in supply-chain management and basic knowledge on DG handling. However, it is important to investigate potential variations in measurement results based on factors such as age and task difficulty or user interface-related issues. Moreover, the participants lacked professional experience, albeit having received education regarding basic DG handling. Therefore, the feasibility of MRSafe can be further illustrated by incorporating a broader spectrum of users such as experienced practitioners. Another limitation of this study is that it only focuses on one category of DG, specifically Class 4, within the MRSafe training program. To provide a more comprehensive safety training system for transporting DG, future research should expand the MRSafe program to include more categories of hazardous materials. By including additional categories, such as Class 1 (explosives and blasting agents), Class 2 (compressed gases), and Class 3 (corrosive substances) [12], the training program can better address the diverse risks and safety considerations associated with different types of DG. This expansion would enhance the effectiveness and relevance of the MRSafe program, ensuring that individuals are adequately trained and equipped to handle a wider range of situations and materials.

## Figures and Tables

**Table 1:** Hardware specification used in the experiment.

Component	Specification
Operating System	Windows 10
CPU	Intel Core i7
GPU	NVIDIA GTX 1060
RAM	16 GB
Storage space	20 GB
Display port	HDMI 2.0
Connectivity	Bluetooth 4.0
Expected framerate	90 Hz

**Table 2:** Overall comparison of the preparation process for both methods.

Performance Indicator	Manual	MRSafe	Improvement
Average duration of the overall training sessions (mins)	8.51	4.78	43.83%
Error rate of the overall training sessions (%)	13.64%	5.76%	7.88%
Error rate of the DG distinguishing process (%)	8.42%	3.55%	4.87%
Average time required for responding to emergency situations (mins)	0.57	0.2	64.91%
Average time required for basic condition checking (mins)	2.94	1.63	44.56%
Average time required for packaging requirements checking (mins)	3.25	2.16	33.54%
Average time required for shipping requirements checking (mins)	1.75	0.79	54.86%

**Table 3:** Survey results.

Survey Questions	Manual (Avg.)	MRSafe (Avg.)
User satisfaction (Scale 1–5)	3.2	4.6
Perceived ease of use (Scale 1–5)	3.0	4.8
Perceived effectiveness (Scale 1–5)	3.1	4.7
User confidence in handling DG (Scale 1–5)	3.3	4.5
User faitigue (Scale 1–5)	3.8	2.5
Behavioral Data		
Number of help requests (measured via support logs)	5	1

**Table 4:** Summary of *t*-test on completion time and user satisfaction.

	Manual		MRSafe			
	Mean	SD	Mean	SD	t	*d*
Completion time	8.51	0.69	4.78	0.62	21.72	3.67
User satisfaction	3.2	1.47	4.6	0.70	7.60	1.28

## Data Availability

Data are contained within the article.
